# Comparison of Toxicity of Benzene Metabolite Hydroquinone in Hematopoietic Stem Cells Derived from Murine Embryonic Yolk Sac and Adult Bone Marrow

**DOI:** 10.1371/journal.pone.0071153

**Published:** 2013-08-05

**Authors:** Jie Zhu, Hong Wang, Shuo Yang, Liqiao Guo, Zhen Li, Wei Wang, Suhan Wang, Wenting Huang, Liping Wang, Tan Yang, Qiang Ma, Yongyi Bi

**Affiliations:** 1 School of Public Health, Wuhan University, Wuhan, Hubei, P.R. China; 2 Hubei Key Laboratory of Allergy and Immune-related Diseases, Wuhan, Hubei, P.R. China; 3 Hubei Biomass-resource Chemistry and Environmental Biotechnology Key Laboratory, Wuhan University, Wuhan, Hubei, P.R. China; 4 China CDC of Xi'An City, Xi'An, Shanxi, P.R. China; 5 Receptor Biology Laboratory, Toxicology and Molecular Biology Branch, Health Effects Laboratory Division, National Institute for Occupational Safety and Health, Centers for Disease Control and Prevention, Morgantown, West Virginia, United States of America; The University of Texas M.D Anderson Cancer Center, United States of America

## Abstract

Benzene is an occupational toxicant and an environmental pollutant that potentially causes hematotoxicity and leukemia in exposed populations. Epidemiological studies suggest an association between an increased incidence of childhood leukemia and benzene exposure during the early stages of pregnancy. However, experimental evidence supporting the association is lacking at the present time. It is believed that benzene and its metabolites target hematopoietic stem cells (HSCs) to cause toxicity and cancer in the hematopoietic system. In the current study, we compared the effects of hydroquinone (HQ), a major metabolite of benzene in humans and animals, on mouse embryonic yolk sac hematopoietic stem cells (YS-HSCs) and adult bone marrow hematopoietic stem cells (BM-HSCs). YS-HSCs and BM-HSCs were isolated and enriched, and were exposed to HQ at increasing concentrations. HQ reduced the proliferation and the differentiation and colony formation, but increased the apoptosis of both YS-HSCs and BM-HSCs. However, the cytotoxic and apoptotic effects of HQ were more apparent and reduction of colony formation by HQ was more severe in YS-HSCs than in BM-HSCs. Differences in gene expression profiles were observed in HQ-treated YS-HSCs and BM-HSCs. Cyp4f18 was induced by HQ both in YS-HSCs and BM-HSCs, whereas DNA-PKcs was induced in BM-HSCs only. The results revealed differential effects of benzene metabolites on embryonic and adult HSCs. The study established an experimental system for comparison of the hematopoietic toxicity and leukemogenicity of benzene and metabolites during mouse embryonic development and adulthood.

## Introduction

Benzene is the simplest aromatic compound and is widely used in industrial and chemical manufacturing. Epidemiological studies and case reports have suggested a close relationship between occupational exposure to benzene and the occurrence of hematotoxicity and various types of leukemia [1], [2]. High-levels of exposure to benzene results in an increased risk of aplastic anemia, pancytopenia, acute myeloid leukemia, and other forms of leukemia in adults [3]–[6].

Childhood leukemia is a major form of cancer in children that develops in the hematopoietic system and is characterized with the production of large amounts of immature white blood cells. The incidence rates of childhood leukemia have been on the rise on an annual basis in recent years. The number of children suffering from leukemia accounts for one third of all children with tumor. Acute lymphoblastic leukemia (ALL) is the most common form of childhood cancer worldwide. The mean annual leukemia incidence per million children is 16.4 in low-income countries, 36.5 in middle-income countries, and 40.9 in high-income countries [7]. The cause and oncogenic development of childhood leukemia and why the incidence increases is largely unknown at the present time.

Although the past research on benzene-induced leukemia has been primarily focused on occupational benzene poisoning and oncogenesis, several recent epidemiological studies have indicated that increased exposure to benzene from the environment is potentially a major cause of childhood leukemia [7]–[10]. For example, Freedman et al. have identified an association between elevated risk of childhood ALL and maternal exposure to interior house painting, which is associated increased exposure of benzene, during the 12 months before the birth of their children [11]. Moreover, several leukemia-associated translocations of DNA were found in embryos. Therefore, it is rational to hypothesize that childhood leukemia may have occurred during the fetal stage of development [12]–[16]. Nonetheless, there is no consensus on whether childhood leukemia is a primary disease, or a disease caused by early exposure to environmental risk factors before or after birth. Overall, these studies suggest that prenatal exposure to benzene may increase hematopoietic system DNA instability, genetic susceptibility to cancer, and exposure to leukemogenic factors in early pregnancy and, thereby, contribute to the increased incidence of childhood leukemia [17]. In these scenarios, benzene or benzene metabolites would have a significant effect on the embryonic hematopoietic system. However, few studies have examined the effects of benzene on embryonic hematopoiesis and childhood leukemia experimentally.

The hematopoietic stem cells (HSC) are responsible for the development of all blood cells. During the development of the embryonic hematopoietic system, placenta, yolk sac and the pre-fusion allantois are recognized as the hematopoietic organs in mammals [18]–[21]. The yolk sac is the major site of embryonic erythropoiesis. Because it is impossible to analyze hematopoietic functions in human fetuses, much of the knowledge on embryonic hematopoiesis comes from studies on mouse embryos [2]. It has been found that the murine embryonic yolk sacs contain the most primitive hematopoietic, pluripotent stem cells——the yolk sac hematopoietic stem cells (YS-HSC) [22]. It is noteworthy to point out that a recent study showed that major HSC in adults are possibly derived from yolk sac precursors [23]. These findings suggest that the murine YS-HSC cells are suitable targets for investigating the toxicity of benzene metabolites on the embryonic hematopoietic system.

Little is known about the mechanism of benzene hematotoxicity and carcinogenicity. We have previously found that the expressions of CYP4F3A and DNA-PKcs were elevated in workers diagnosed with benzene poisoning by microarray analysis of peripheral mononuclear blood cells. In K562 and HL-60 cells, CYP4F3A and DNA-PKcs were shown to be induced by benzene metabolites at both protein and mRNA levels [24]–[27]. CYP4f3A encodes the leukotriene B_4_ ω-hydroxylase in human polymorphonuclear leukocytes. The CYP4f18 is the murine ortholog of the human CYP4f3 [28]. DNA-PKcs is the catalytic subunit of the nuclear DNA-dependent serine/threonine protein kinase that plays a critical role in the non-homologous end joining (NHEJ) pathway of DNA double strand (DSB) break repair [29]. Whether CYP4F18 and DNA-PKcs can be induced by benzene and metabolites in murine adult and embryonic hematopoietic stem cells remains unknown.

The goal of the present study was to investigate the toxicity of hydroquinone (HQ), a major metabolite of benzene in both humans and animals, on YS-HSC in mice and to determine whether YS-HSC are more susceptible to HQ effects than BM-HSC as a possible contributing mechanism to childhood leukemia. We measured the effects of HQ on the colony formation, cell proliferation and apoptosis of the HSCs. Expression of the Cyp4f18 and DNA-PKcs were measured to explore the effect of HQ at the level of gene expression and protein.

## Materials and Methods

### Ethics Statement

All experiments were approved by the Institutional Animal Care and Use Committee of Wuhan University (No. 10061), and the animal procedures were performed in accordance with institutional and national guidelines. All surgery was performed under anesthesia with ketamine and lidocaine, and all efforts were made to minimize suffering.

### Isolation and proliferation of hematopoietic stem cells from murine embryonic yolk sac and adult bone marrow

Special pathogen free (SPF) Kunming mice were purchased from the Central Animal Facility of Wuhan University. Kunming mice were original from Swiss mice and were brought to Kunming, China, from the Indian Haffkine Institute in 1944. This mice line has been widely utilized in pharmacological, toxicological, medicinal and biological research and testing because of its' high disease resistance, good adaptive capacity, high breeding coefficient and good survival rate.

For isolation of BM-HSC, 8–10 weeks old Kunming male mice were used. Murine bone marrow cells from femur and tibias were collected by flushing the shaft with a buffer using a syringe and a 26G needle. The cells were filtered through a 40 µm nylon mesh (BD Biosciences). The cells were first depleted of lineage positive (Lin^+^) cells using magnetic columns and then was positively selected using anti-Sca1 antibodies and anti-murine CD117 antibodies conjugated to mini-magnetic beads (Miltenyi Biotec, Inc., Auburn, CA, http://www.miltenyibiotec.com) according to the manufacturer's instructions. Up to 4.5×10^6^ of lineage^−^ CD117^+^ Sca1^+^ cells can be isolated from two murine cell suspensions.

For isolation of YS-HSC, the uterine horns were collected from a pregnant female on 8–10 days post conception. The endometrial tissues were removed from around each embryo conceptus under the microscope. The placental tissue was removed to reveal the embryo encased in the yolk sac by using the forceps to cut the embryo-encased yolk sac away from the placenta at their junctures. The yolk sac containing vitelline arteries and a portion of the umbilical cord can be gently teased away from the embryo and set aside on ice for dissociation. The yolk sacs were placed in separate tubes. The tissues were pelleted by a brief spin and were resuspended in 0.25% collagenase in PBS with 20% FBS, with enough volume to adequately cover the tissues. The tissues were incubated at 37°C for 40 min for yolk sacs. At the end of the incubation, the tissues were dissociated into single cell suspensions by gently pipetting up and down. The cells were filtered through a 40 µm nylon mesh. The cells was first depleted of lineage positive (Lin^+^) cells using magnetic columns and was then positively selected by using anti-murine CD117 antibodies conjugated to mini-magnetic beads (Miltenyi Biotec, Inc., Auburn, CA, http://www.miltenyibiotec.com). Up to 3.2×10^6^ of lineage^−^ CD117^+^ cells were isolated from eight yolk sac cell suspensions.

Isolated HSC cells were cultured at 37°C with 5% humidified CO_2_ in the StemSpan™ Serum-Free Expansion Medium (Stem Cell Technologies, Inc, Vancouver, BC, Canada) in polystyrene 6-well plates with the addition of cytokines SCF (100 ng/ml), IL-3 (20 ng/ml), and Flt-3 (100 ng/ml) (Peprotech USA).

### Histology and immunohistochemistry

Paraformaldehyde fixed embryonic yolk sac tissues were embedded in paraffin and sectioned at 5 µm thickness using standard procedures performed at a Pathology Laboratory, Wuhan University School of Medicine. Samples were embedded in paraffin and at least two sections per tissue were prepared and stained with hematoxylin and eosin. After dewaxing and hydration, the paraffin sections were rinsed three times with PBS (0.01 M, pH 7.5) each for 3 min. Antigen retrieval was performed according to the requirements of each antibody. Each slide was incubated for 10 min at room temperature in a 50 µl peroxidase blocking solution (UltraSensitive™SP Kit, Maixin.Bio) to block endogenous peroxidase activities. The slides were then washed three times with PBS and were incubated for 10 min in 50 µl of a normal non-immune serum (UltraSensitive™SP Kit, Maixin.Bio) at room temperature. Staining for CD41 was performed using rabbit polyclonal antibodies (Biolegend USA) incubated at 4°C overnight. After washing in PBS, the slides were incubated in 1∶150 dilution of appropriate secondary antibodies conjugated to biotin rabbit anti-rat antibodies (Boster, Wuhan, China), followed by incubation with Stretavidin-Peroxidase (UltraSensitive™SP Kit, Maixin.Bio) for 10 min. The slides were stained with DAB and processed for mounting with neutral gums.

### Immunophenotyping

After separation of yolk sacs using the CD117 MicroBeads, the cells were washed and fluorescently stained with mouse CD117 antibodies conjugated with PE (#130-0991-730) by incubation for 10 min in the dark on ice. The cells were washed and resuspended in PBS containing 0.5% bovine serum albumin (BSA). Flow cytometric analysis was carried out on a flow cytometer (Epics Altra II, Beckman, USA).

### Colony-forming unit (CFU) assay

A cell suspension of 5×10^4^ cells/mL was used. About 0.3 mL of cells was added to 3 ml of the MethoCult® Medium containing 0, 1.25, 2.5 and 5.0 µM of HQ supplemented with SCF, IL-3, IL-6, and Epo (Stem Cell Technologies, Inc, Vancouver, BC, Canada) for duplicate cultures. The tubes were votexed to ensure all cells and components are thoroughly mixed. The tubes were allowed to stand for 5 min to allow bubbles to dissipate. The medium was dispensed into culture dishes, 1.1 ml per 35 mm dish. A needle was placed below the surface of the solution to gently depress the plunger and expel the medium completely until no air space is visible. Two dishes were placed into a 100 mm petri dish. A third, uncovered 35 mm dish containing 3 ml of sterile water was placed in to maintain humidity and minimize contamination during culture and handling. The dishes were cultured in an incubator maintained at 37°C with 5% CO_2_ and 95% humidity.

### Cytotoxicity assays

Cell viability and cell count were evaluated by using the CCK-8 and Trypan blue exclusion assays after 24 h of HQ exposure. For the CCK-8 assay, 100 µl of cell suspension (5,000 cells/well) were seeded in a 96-well plate that was preincubated for 24 h at 37°C, 5% CO_2_. Ten µl of various concentrations of substances to be tested was added to the plates. The plate was incubated for 24 h in the incubator. Ten µl CCK-8 was added into each well and incubation was continued for another 1 h. Absorbance was measured at 450 nm using a microplate reader. For the Trypan blue exclusion assay, about 1×10^6^ cells were seeded onto a 60 mm culture dish and then, were treated with HQ. Cells were scraped, pelleted and resuspended in PBS. Equal volumes of cell suspension and 0.4% Trypan blue were mixed. Viable cells were counted under a microscope and cell viability was expressed as the viable cell numbers/total cell numbers multiplied by 100.

### Apoptosis analysis

The Annexin V/Propidium Iodide (PI) kit (KeyGEN Biotech, Nanjing, China) were used to evaluate apoptosis by flow cytometer according to the manufacturer's protocol (Epics Altra II, Beckman, USA) performed at Center for Medical Research at Wuhan University School of Medicine. Briefly, cells were washed twice with cold PBS and resuspended in 500 µl binding buffer, and were then stained with 5 µl Annexin V-FITC and 10 µl PI. The cells were incubated for 10 min at room temperature in the dark and analyzed in a Beckman flow cytometer. Percent of cells undergoing early apoptosis (Annexin V-positive and PI-negative) and late apoptosis (Annexin V-positive and PI- positive) were combined to derive the percentages of total apoptotic cells.

### Quantitative real–time PCR

Total RNA was extracted using the TRIzol reagent (Invitrogen, Carlsbad, CA). One µg of total RNA was reverse-transcribed to complementary DNA using the All-in-One First Strand cDNA Synthesis Kit (GeneCopoeia, USA). Quantitative real–time PCR was performed on the ABI StepOne plus Real-Time PCR System, using the All-in-One qPCR Mix (GeneCopoeia, USA). The PCR amplification conditions were as follows: 95°C for 10 min (Initial denaturation); 40 cycles of 95°C for 10 seconds (denaturation); 55°C for 20 seconds (annealing) and 72°C for 30 seconds (extension). The relative expression of target genes was calculated using the 2^−ΔΔCt^ method. β-actin was used as an internal control. The primers used were as follows: DNA-PKcs forward 5′-TGTCACAAGAGGAGAAAGTGGC-3′ and reverse 5′-TGTACATTAGCACATAGGCTCC-3′; CYP4f18 forward 5′-AGAGCCTGGTGCGAACCTT-3′ and reverse 5′-TGGAATATACGGATGACTGG-3′; β-actin forward 5′-AACAGTCCGCCTAGAAGCAC-3′and reverse 5′- CGTTGACATCCGTAAAGACC-3′.

### Immunofluorescence

For the detection of γ-H2AX, cells (1.2–1.5×10^6^/ml) were seeded into 60 mm culture dishes and treated with HQ for 6 h. Cells were washed once with PBS for 5 min, and then were paraformaldehyde-fixed and blocked with BSA prior to incubation with anti- γH2A.X (phospho S139) antibody (ab11174, abcam) for 45 min at 37°C. An Alexa-Fluor ® 488 conjugated goat anti-rabbit antibody (Jackson ImmunoResearch Laboratories, West Grove, PA) was used as the secondary antibody at a dilution of 1∶500 in PBS incubated for 30 min at 37°C. Cells were washed with PBS and were counterstained with 3 µg/ml PI (Sigma, MO) in PBS for 5 min. Fluorescence was detected under a confocal microscope.

### Immunoblotting

Cells (3.0–3.5×10^6^/ml) were seeded into 60-mm culture dishes and treated with HQ for 24 h. Cells were washed twice with ice-cold PBS and then lysed in a lysis buffer (2 mM sodium orthovanadate, 50 mM NaF, 20 mM HEPES, pH 7.5, 150 mM NaCl, 1.5 mM MgCl_2_, 5 mM sodium pyrophosphate, 10% glycerol, 0.2%Triton X-100, 5 mM EDTA, 1 mM PMSF, 10 µg/ml leupeptin, and 10 µg/ml aprotinin) on ice for 30 min. Insoluble materials were pelleted at 14,000×g for 20 min at 4°C. Whole-cell protein was mixed with the SDS sample buffer, and was boiled for 5 min. Equal amounts of protein (30 µg) were electrophoresed on 10% SDS/PAGE and were electroblotted onto PVDF membranes and identified with anti-Cyp4f3 polyclonal antibodies (Abnova, Taipei, Taiwan) (the antibody recognizes the murine ortholog Cyp4F18 of human Cyp4F3 by immunoblotting), anti-p53 polyclonal antibodies (ABclonal, USA) and anti-GAPDH polyclonal antibodies (Bioworld Technology, USA). Secondary goat anti-mouse IgG–HRP (Bioworld Technology, USA) or goat anti-rabbit IgG–HRP (Bioworld Technology, USA) was used according to the species of primary antibody. Chemiluminescence was performed using SuperSignal West Dura (Pierce) and the FluorChem 8900 visualization system (Alpha Innotech). The relative density of detected signals was analyzed by using the image analysis software (Image 1.56, NIH).

### Statistical analysis

SPSS 17.0 was used for data analysis. Data were expressed as means ± SDs. The statistical analysis was determined by one-way analysis of variance (ANOVA) followed by Least Significant Difference test (LSD) and Dunnett's T3. Differences of total colonies forming units between BM-HSC and YS-HSC in the HQ = 1.25 µM groups were performed using independent two-tailed *t* test. *P*<0.05 was considered statistically significant.

## Results

### Isolation of HSCs from murine yolk sacs

**Figure 1:**
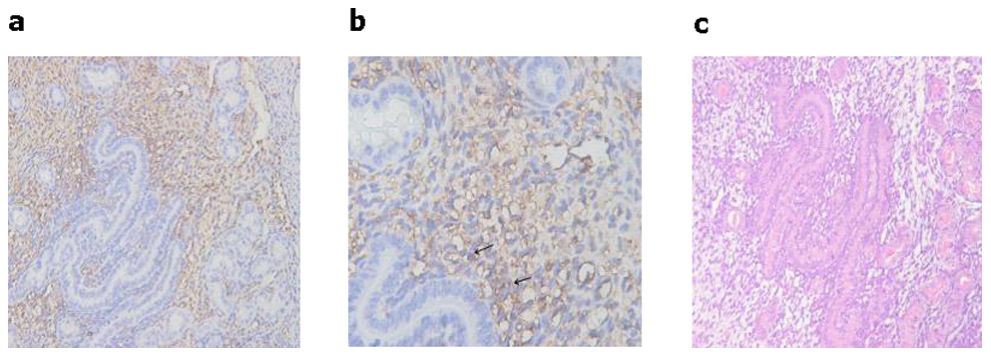
Identification of YS-HSCs. (a, b, and c) Immunohistochemistry staining of CD41 in E9 murine embyo yolk sacs. The arrows refer to cells expressing CD41 in E9 murine embryo yolk sacs. Magnifications: (a) 200×; (b) 400×; and (c) A representative section of H & E staining of the E9 murine embryo yolk sacs (magnification of 200×).

**Figure 2:**
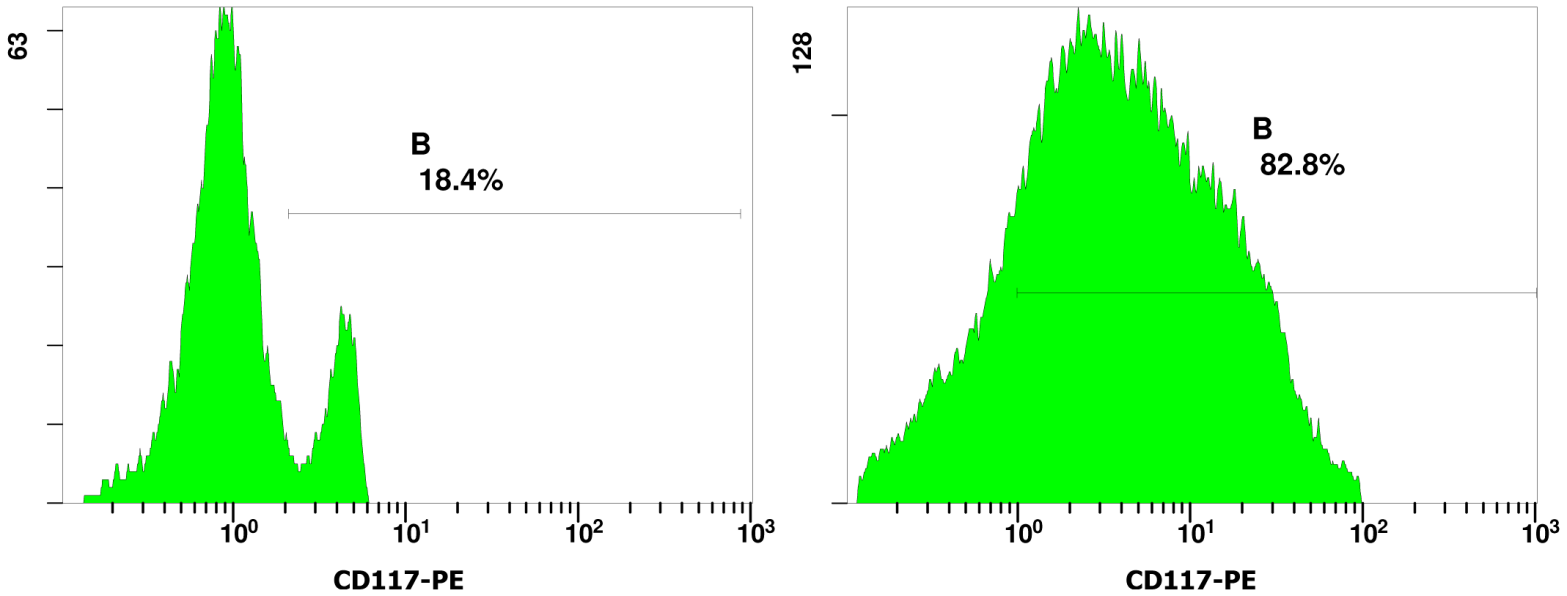
Immunophenotyping of CD117^+^ yolk sac cells. Representative flow cytometric data from more than three independent analyses were shown. The cells were fluorescently stained with CD117-PE. To show the CD117^+^ cell purity, CD117^+^ yolk sac cells before separation were shown on the left panel; CD117^+^ yolk sac cells after separation by using the anti-murine CD117 antibodies conjugated to mini-magnetic beads were shown on the right panel.

The HSCs in murine yolk sacs were identified by their expression of CD41 and CD117 on the cell surface. CD41 is a specific cell surface marker of nascent HSCs [30]–[32]. CD117 (c-kit) has also been identified as a marker on HSCs [33], [34]. Figure 1 shows the immunohistochemistry staining of CD41 positive hematopoietic progenitor cells from murine yolk sacs. CD41-expressing cells were found to be present in the E9 (embryonic day 9) murine yolk sacs. Expression of the hematopoietic stem cell antigen maker CD117 on the surface of sorted yolk sac cells were analyzed by flow cytometry. Figure 2 illustrates one typical experiment from a total of three experiments. From murine yolk sac cells containing about 19.35±6.29% (n = 4) CD117^+^ cells, the CD117 positive cells in the enriched fraction typically account for up to 78.38±9.34% (n = 4) (Fig. 2).

### Effect of HQ on colony-forming units of YS-HSC and BM-HSC

**Figure 3:**
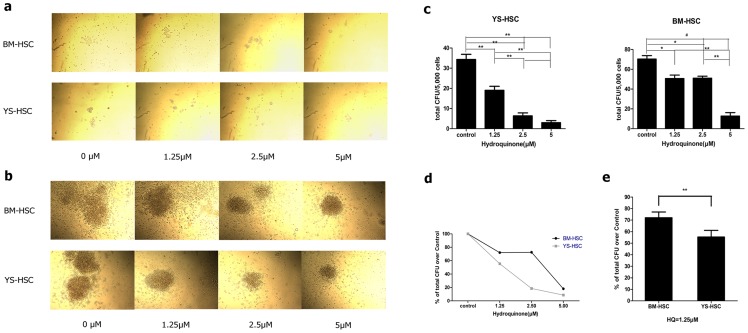
Effect of HQ on CFU of YS-HSC and BM-HSC after 7 days of culture in vitro. Five thousand cells were sorted directly into the methylcellulose medium containing 0, 1.25, 2.5, or 5.0 µM hydroquinone supplemented with SCF, IL-3, IL-6, and Epo. Colony numbers were counted on day 7. Each experiment was done in triplicates. Quantitative data represents the average number of colonies per dish. (**a**) Morphology of CFU-E/BFU-E of BM-HSC and YS-HSC after 7d culture. (**b**) Morphology of CFU-GM of BM-HSC and YS-HSC after 7d culture. (**c**) CFU numbers of BM-HSC and YS-HSC. (**d**) Comparison of total CFU as percent of control between BM-HSC and YS-HSC. (**e**) Comparison of total CFU as percent of control between BM-HSC and YS-HSC treated with HQ at 1.25 µM. Data represent means ± SDs of three or more separate experiments (*, *P*<0.05; **, *P*<0.01; and #, *P*<0.001). CFU-GM includes CFU-granulocyte (CFU-G), CFU-macrophage (CFU-M), and CFU-granulocyte macrophage (CFU-GM). CFU-E represents colony-forming unit-erythroid. BFU-E represents burst-forming unit-erythroid.

The colony sizes of CFU-GM and CFU-E/BFU-E and the total CFU numbers decreased upon treatment with increasing concentrations of HQ in both BM-HSC and YS-HSC (Figure 3). However, the decrease was markedly more apparent in YS-HSC than BM-HSC. At 5.0 µM of HQ, there was no typical erythrocyte colony formation in HQ-treated YS-HSC, but colony formation in HQ-treated BM-HSC was still observed. Quantification of CFUs expressed as percent of controls confirmed that HQ reduced the CFUs in concentration-dependent manners in both BM-HSC and YS-HSC; however, the reduction in YS-HSCs was significantly faster as HQ concentration increases than BM-HSC (Figure 3c and d). It is noteworthy to point out that no significant differences were detected in the total CFU numbers of BM-HSC between the 1.25 and 2.5 µM groups (Figure 3c and d). These results indicated that HQ has significantly higher toxicity on the differentiation and proliferation of embryonic HSC than adult bone marrow HSCs.

### Cytotoxicity of HQ on YS-HSC and BM-HSC

**Figure 4:**
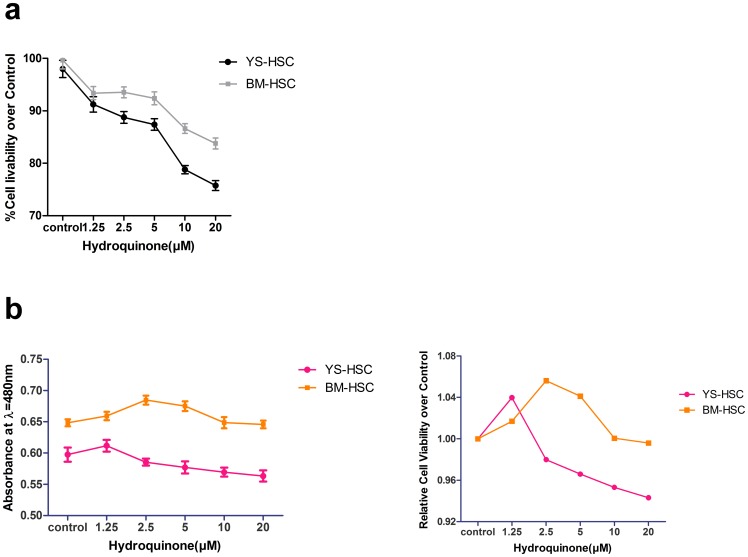
Comparison of cytotoxicities of HQ on BM-HSC and YS-HSC. Cells were exposed to increasing concentrations of HQ after 24 h liquid culture. (a) Cell viability was measured by the trypan blue exclusion assay. The initial cell amount used was 1×10^5^/ml. Relative cell viability was represented as the percentage of viability of treated group over the control. (b) Cell viability was determined by the CCK-8 assay. Left: Viability of BM-HSC and YS-HSC; right: Relative viability of BM-HSC and YS-HSC over control. Data represent means ± SDs (n = 3).

CCK-8 and trypan blue assays were conducted to determine the cytotoxicity of HQ on HSCs. Cell viability was measured by trypan blue exclusion under microscope after the cells were treated with hydroquinone for 24 h. There was a negative correlation between HQ concentrations and the cell survival rates both in BM-HSC and YS-HSC (Figure 4a). However, in comparison with the bone marrow HSCs, reduction in the viability of YS-HSC was more obvious than that of BM-HSC. Similar results were obtained by using the CCK-8 assay (Figure 4b). These findings indicate that, within the HQ concentration range examined, the cytotoxicity of HQ on YS-HSC was more noticeable than on BM-HSC.

### HQ-Induced Apoptosis in BM-HSC and YS-HSC

**Figure 5:**
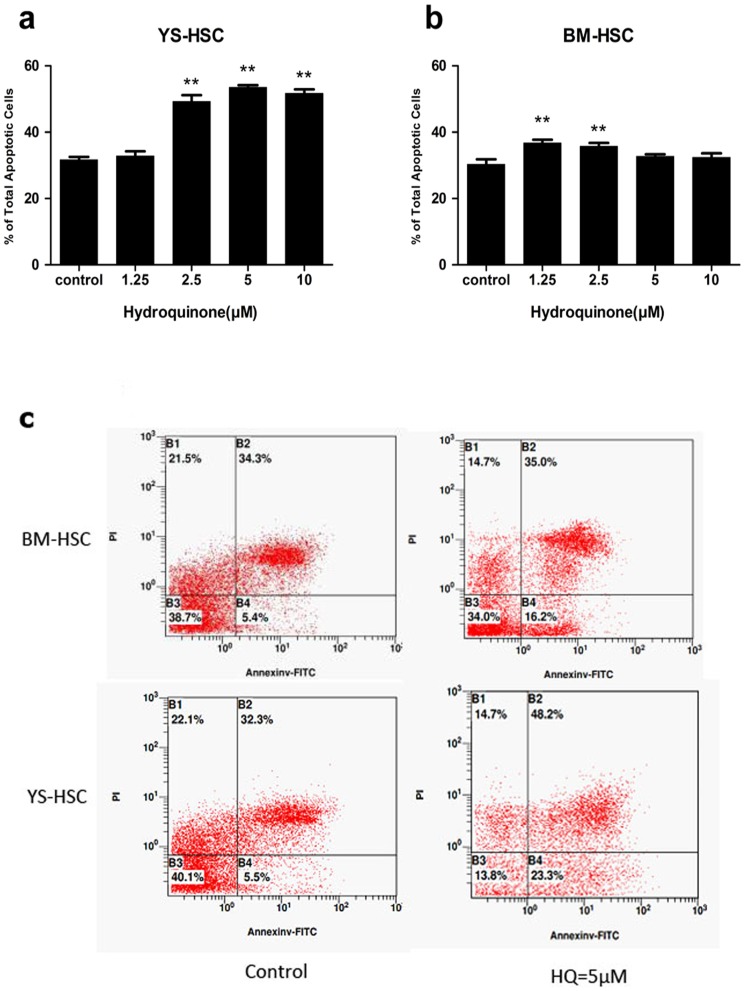
Comparison of apoptotic effects of HQ on BM-HSC and YS-HSC. Cells were exposed to various concentrations of HQ after 24 h liquid culture and were stained with Annexin V and PI. Apoptosis was determined using flow cytometry as described in Materials and Methods. Illustrated is a representative of three separate experiments. (a) Percent of total apoptotic cells of YS-HSC. (b) Percent of total apoptotic cells of BM-HSC. (**P<0.01, compared to control group values). (c) A representative flow cytometry data showing apoptotic effects of HQ on BM-HSC and YS-HSC at the 5 µM concentration.

Since differential cytotoxic effects were observed between BM-HSC and YS-HSC following treatment with HQ, we evaluated the level of apoptosis in the cells as a possible mechanism for the observed differences. BM-HSC and YS-HSC were treated with HQ at increasing concentrations. The percentages of cells undergoing early apoptosis (annexin V positive and PI negative) and late apoptosis (annexin V positive and PI positive) were combined as the total apoptosis as described under Materials and Methods. HQ increased apoptosis in BM-HSC cells at 1.25 and 2.5 µM concentrations slightly compared with the control (Figure 5b). On the other hand, HQ induced large increases in apoptosis at concentrations of 2.5, 5, and 10 µM in YS-HSC (Figure 5a). The increases in YS-HSC were markedly more apparent compared with those of BM-HSC treated with the same concentrations of HQ (Figure 5c). These results indicated that, within the HQ concentration range examined, the apoptotic effect of HQ on YS-HSC was significantly larger than on BM-HSC.

### Induction of p53 protein by HQ

**Figure 6:**
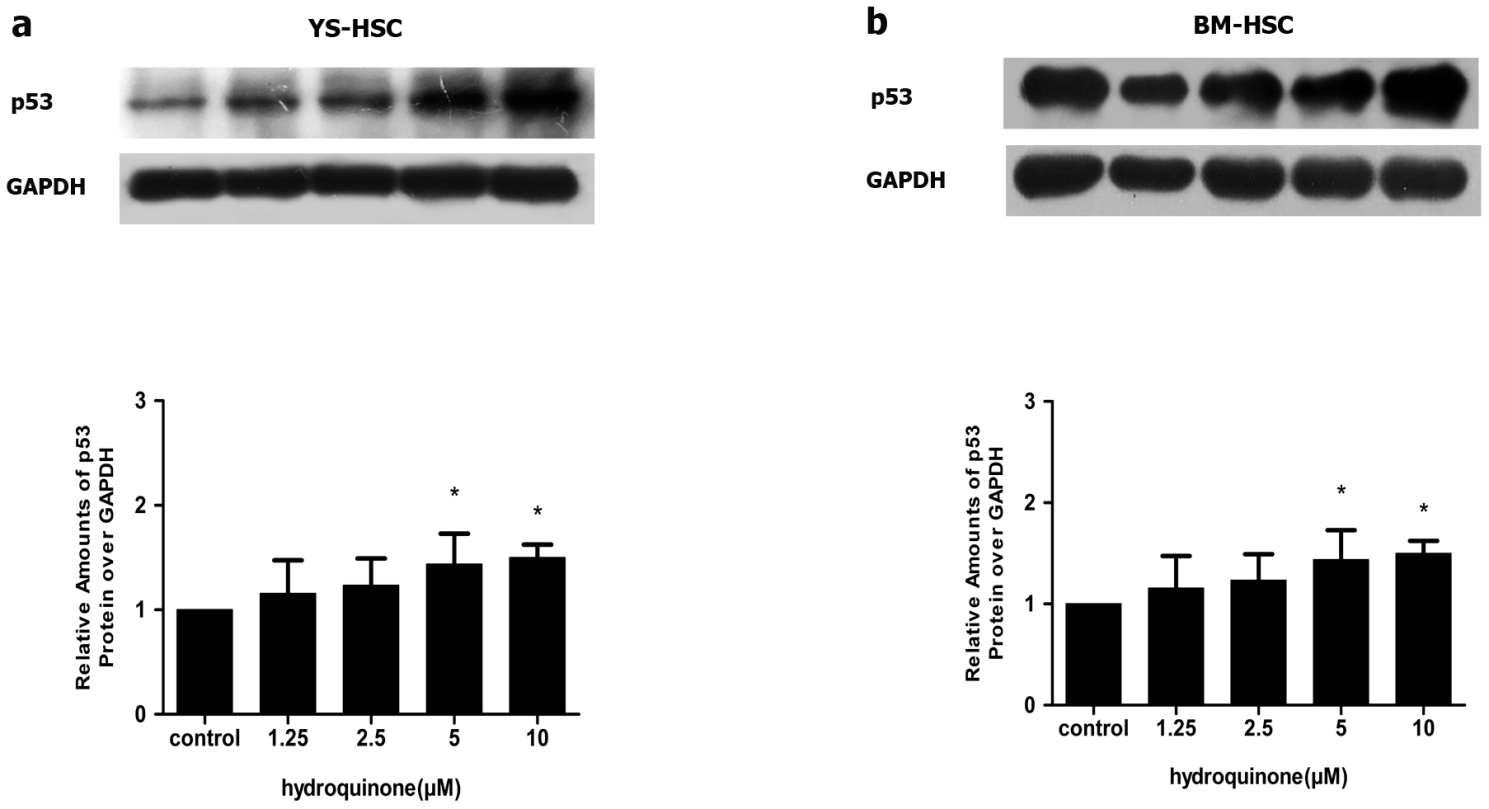
Induction of p53 by HQ. YS-HSC (a) and BM-HSC (b) were treated with increasing concentrations of HQ for 24 h. Total cell lysates were analyzed by immunoblotting with antibodies against p53. Expression of the GAPDH protein was used as a loading control. Upper panels: representative blotting results. Densitometry of the immunoblotting results was shown in the lower panels. Values are presented as means ± SDs (n = 3–5). * *P*<0.05.

The p53 protein plays an important role in apoptosis and the protein is induced in apoptotic cells. To further verify the apoptotic effect of HQ, p53 protein expression was measured by immunoblotting after the cells were treated with increasing concentrations of HQ. Figure 6 showed that the p53 protein levels increased significantly after 24 h of incubation with HQ in concentration-dependent manners in both YS-HSC and BM-HSC. However, the basal level of p53 was much lower in YS-HSC than in BM-HSC and induction by HQ was significantly more apparent in YS-HSC and BM-HSC. The reason why the basal level of p53 in BM-HSC was higher than that of YS-HSC is unclear at the present.

### Induction of Cyp4f18 in murine YS-HSC and BM-HSC

**Figure 7:**
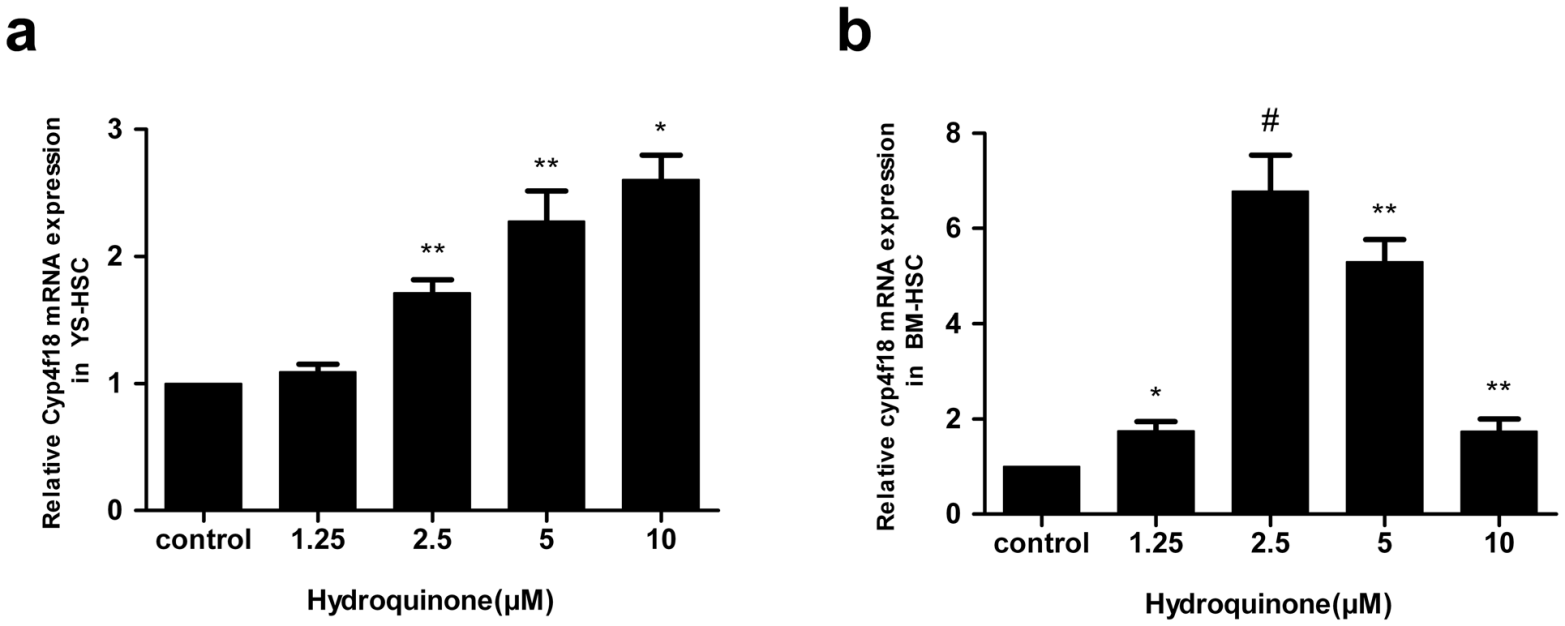
Effect of HQ on Cyp4f18 mRNA expression. Expression of Cyp4f18 mRNA in YS-HSC (a) and BM-HSC (b) was measured by quantitative real–time PCR. The relative expression of target genes was calculated using the 2^−ΔΔCt^ method. Data represent means ± SDs of at least three individual experiments (*, *P*<0.05; **, *P*<0.01; and #, *P*<0.001; compared to control group values.).

**Figure 8:**
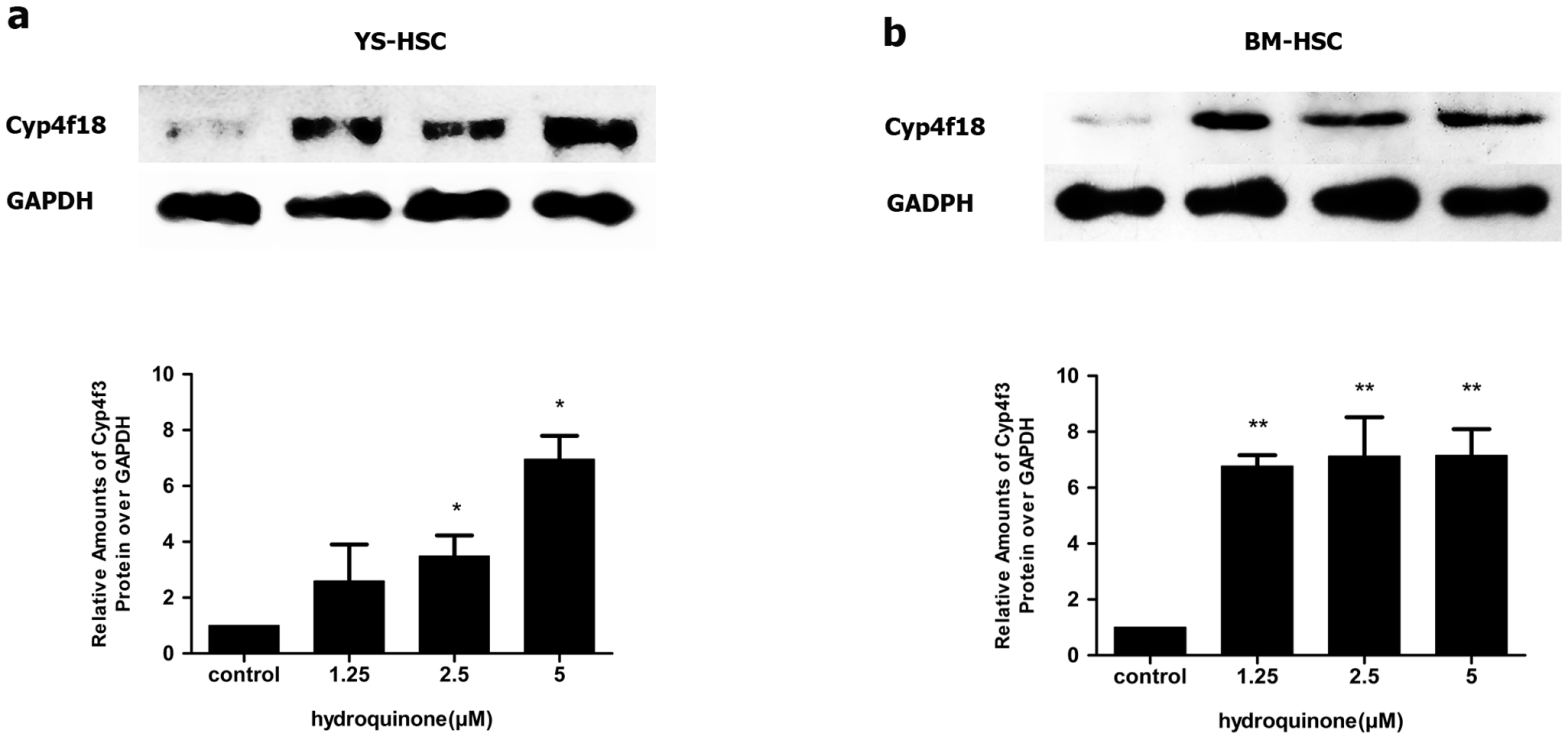
Effects of HQ on Cyp4F18 protein expression. Immunoblot analysis of Cyp4f18 proteins on HQ-treated YS-HSC (a) and BM-HSC (b) cells. Immunoblotting of Cyp4F18 was shown in the upper panels. GAPDH was measured as loading control. Densitometry of immunoblot results was shown in the lower panels. Values are presented as means ± SDs (n = 3–5). *, *P*<0.05; **P<0.01; compared with controls.

The murine Cyp4f18 gene is an ortholog of the human CYP4F3 gene that is induced by benzene metabolites in human blood cells. To examine the effect of HQ on gene expression in HSC cells, the mRNA levels of Cyp4f18 were determined by quantitative real–time PCR in BM-HSC and YS-HSC. As shown in Figure 7a, HQ induced the expression of Cyp4f18 mRNA expression in a concentration-dependent manner in YS-HSC. Induction was seen at 2.5 µM and was highest at 10 µM (<3-fold). In BM-HSC, Induction was dramatically higher in cells treated with 2.5 µM of HQ (>6-fold, compared with control, Figure 7b). However, induction was decreased in higher concentrations of HQ. Therefore, induction of Cyp4f18 mRNA by HQ exhibited different concentration-dependence characteristics in YS-HSC and BM-HSC. The expression and induction of Cyp4F18 proteins in YS-HSC and BM-HSC were examined by immunoblotting. Both YS-HSC (Figure 8a) and BM-HSC (Figure 8b) showed large inductions of the Cyp4F18 protein by HQ.

### Induction of DNA-PKcs and DNA double strand break in BM-HSC

**Figure 9:**
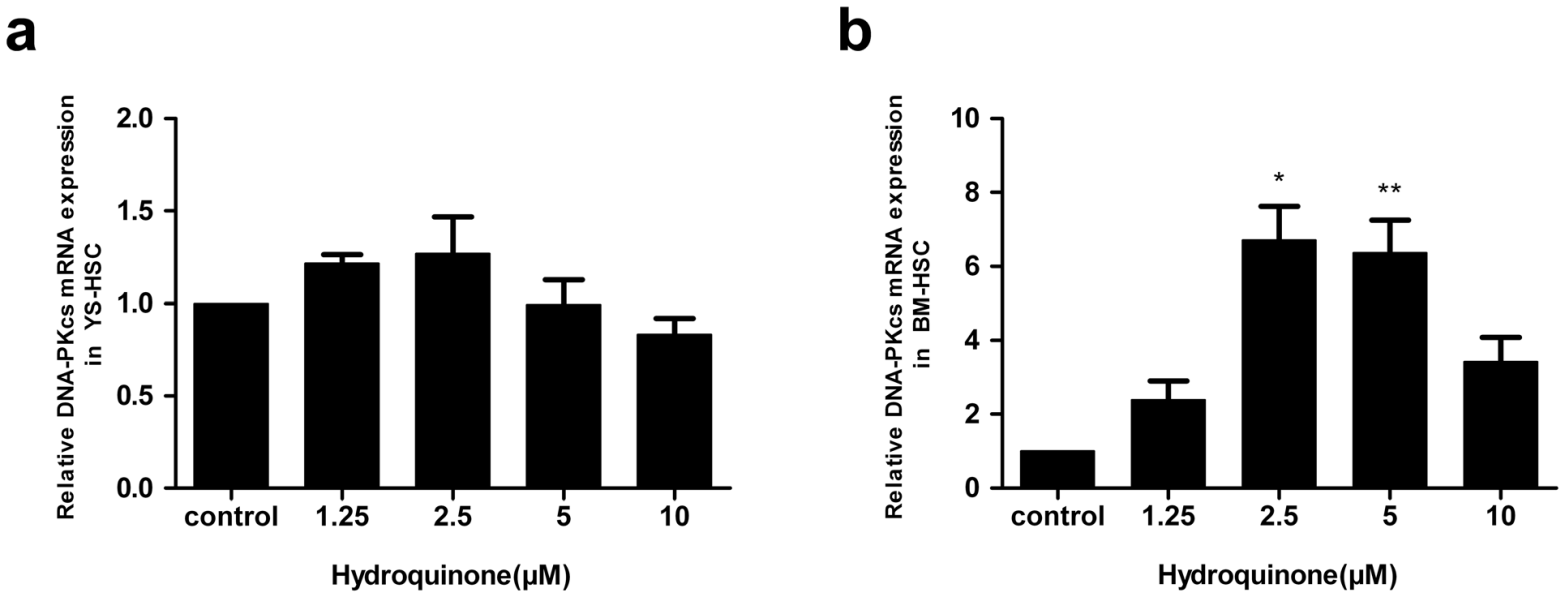
Effect of hydroquinone on DNA-PKcs mRNA expression. YS-HSC (a) and BM-HSC (b) cells were treated with HQ at increasing concentrations and expression of DNA-PKcs mRNA was measured by quantitative real–time PCR. The relative expression of target genes was calculated using the 2^−ΔΔCt^ method. Data represent means ± SDs of at least three individual experiments (*, *P*<0.05; **, *P*<0.01; and #, *P*<0.001; compared to control group values.).

Benzene and its metabolites affect the expression of DNA-PKcs and DNA double strand break repair as indicated by induction of γ-H2AX foci formation in blood cells. Therefore, we measured the effect of HQ on DNA-PKcs expression and γ-H2Ax formation in the HSC cells. DNA-PKcs gene expression levels exhibited no statistically significant differences among control and all HQ treated groups in YS-HSC (Figure 9a). On the other hand, DNA-PKcs mRNA levels were significantly higher in BM-HSC cells treated with HQ at 2.5 and 5 µM (Figure 9b).

**Figure 10:**
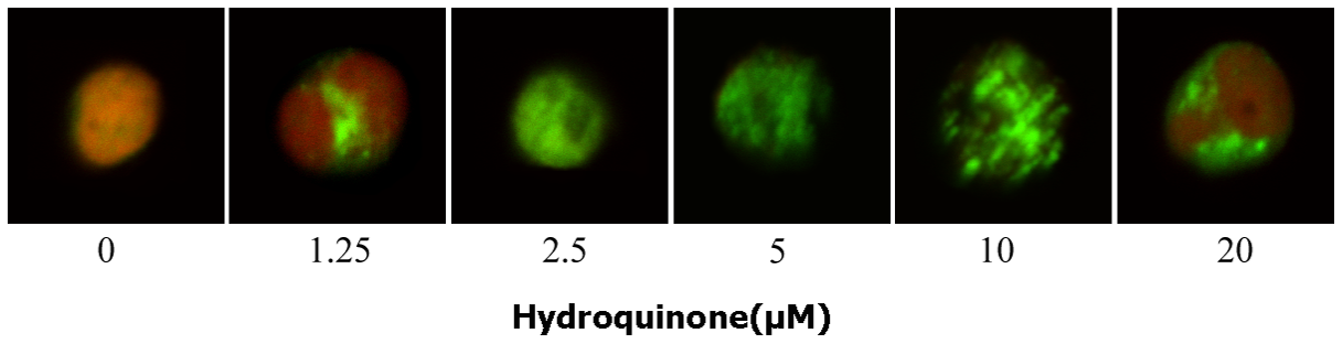
DSB repair induced by HQ in BM-HSC. Representative microscopic fluorescence images of γ-H2AX foci accumulation after HQ treatment for 6 h. Nuclei were stained using PI and Al-exa488-labeled secondary antibodies, and were examined by confocal microscopy (oil, 200×). The experiments were repeated for three more times. Green fluorescence indicates formation of γ-H2AX foci.

The finding that HQ induced DNA-PKcs in BM-HSC prompted us to examine whether HQ induces DNA double strand break repair in BM-HSC. Formation of γ-H2AX foci is characteristic of DNA double strand break repair in cells. Exposure of BM-HSC cells to HQ for 6 h led to concentration-dependent formation of γ-H2AX foci (Fig. 10).

## Discussion

The present study was designed to compare the toxicities of the benzene metabolite hydroquinone on hematopoietic stem cells derived from murine adult bone marrow and embryonic yolk sac in order to explore a potential role of benzene and metabolites in the development of childhood leukemia. The experiment was based on the assumption that, in children with leukemia, oncogenic lesions of the hematopoietic system began to appear during the fetal development and were accumulated as a continuation of the pregnancy, which ultimately results in the development of leukemia after birth. Benzene and metabolites may induce a number of chromosomal aberrations including mitotic recombination, DNA strand breaks, chromosomal translocations, and aneuploidy in hematopoietic stem or progenitor cells. These lesions would disrupt the normal process of self-renewal, proliferation and differentiation of HSC, but confers HSC with an unlimited proliferative capacity, consequently causing the arise of AML leukemia stem cells (or leukemic clones) and leukemogenesis [4], [35]–[39]. Thus, the study on the toxic effects of benzene exposure on adult and embryonic hematopoietic stem cells may reveal new mechanistic aspects of the pathogenesis of leukemia, especially childhood leukemia.

It has been demonstrated that YS-HSC have lymphoid, myeloid and erythroid precusor potentials [40]–[42]. By following the emergence of hematopoietic stem cells in murine yolk sac [43], [44], a dominant YS-HSC in day 8.5–9.5 embryos was isolated as the main source of embryonic hematopoietic stem cells. Currently, the general method for isolation and purification of HSC was based on the specific antigen markers on the cell surface. Isolation of hematopoietic stem cells derived from bone marrow of adult mice with the KSL (C-kit, Sca-1, lineage) phenotype is widely used in the purification of murine HSC. However, for embryonic hematopoietic stem cell sorting, there is no generally accepted sorting method available. Compared to other hematopoietic stem cells such as those derived from fetal liver and bone marrow, the murine yolk sac hematopoietic stem cells express no stem cell antigen (Sca-1), but express c-kit (CD117), the receptor of Throm-bopoietin (TPO) [44]–[47]. Given the stability and specificity of cell surface antigen expression, c-kit and lineage were used as the sorting markers for YS-HSC isolation.

The colony-forming cell (CFC) assays are generally used to quantify multi-potential progenitor and restricted progenitor cells. Under an optimal condition, immature progenitors develop into larger colonies containing cells of two or more lineages, whereas mature progenitors develop into colonies containing cells of only a single lineage. The CFC assays have become the benchmark functional characterization to assess the ability of various hematopoietic cell types to divide and differentiate since its introduction over four decades ago [48]. It is also used as an alternative method to animal models for the evaluation of the efficacy or lineage-specific toxicity of drugs and environmental chemicals on human and animal hematopoietic cells [49]. Studies have found that benzene exposure suppresses hematopoietic progenitor cell differentiation of multilineage colony forming unit (CFU-GEMM), colony forming unit (CFU-GM) and blast forming unit (BFUE/CFUE) in mice [50]. Hydroquinone exposure results in lineage-specific toxicities in both bone marrow and yolk sac hematopoietic stem cells by the CFC assay. At the concentration of 1.25 µM, hydroquinone caused more toxic effects on YS-HSC than that of BM-HSC, suggesting that the progenitor differentiation and proliferation of embryonic hematopoietic stem cells are more likely to be affected by hydroquinone than adult hematopoietic stem cells.

During HSC self-renewal, mature progenitor cells may arise from HSC in culture. Such mature cells may not be easily distinguished from HSC, but the existence of the cells may complicate the toxic effect of hydroquinone on the proliferative capability of HSC because mature cells are less proliferative. The CCK-8 assay was designed to determine the number of viable primitive cells in the cell proliferation and cytotoxicity assays without being affected by the presence of mature progenitor cells. Our study revealed that both the cytotoxicity and the apoptosis-inducing effect of HQ on YS-HSC were more noticeable than on BM-HSC. The findings support the notion that cumulative toxic effect is likely to occur in embryonic hematopoietic stem cells, which leads to damage on the embryonic hematopoietic system.

We have previously reported that CYP4F3A and DNA-PKcs mRNAs were elevated in workers diagnosed with benzene poisoning. We also found that CYP4F3A and DNA-PKcs were induced by benzene metabolites hydroquinone and phenol in HL-60 and K562 cells at both mRNA and protein levels. Expression of CYP4F3A siRNA was also found to inhibit HL-60 cell proliferation [24]–[26]. The murine Cyp4F18 is a functional ortholog of the human Cyp4F3A and it is the main regulatory protein in LTB4 metabolism in murine polymorphonuclear leukocytes. Cyp4F18 deactivates LTB-4 and, thereby, contributes to suppression of inflammation [28]. Whether Cyp4f18 can be induced by benzene and metabolites in murine hematopoietic stem cells has not been investigated. We observed a greater than 6-fold increase in the mRNA levels by HQ in BM-HSC, whereas a smaller than 3-fold induction of this gene was observed in YS-HSC (Fig. 7a, b). Induction of Cyp4f18 inhibits the recruitment of neutrophils, eosinophils and CD4+ effector T-cells into tissues during inflammation [51].

The DNA-PKcs, together with the Ku70/Ku80 heterodimer forms the biologically active DNA-PK complex that plays a key role in the repair of DNA double-strand breaks through the non-homologous end-joining (NHEJ) pathway. We found that DNA-PKcs can be induced by HQ in BM-HSC but not in YS-HSC, suggesting that the nonhomologous end-joining pathway was not induced in YS-HSC. Benzene and metabolites can cause DNA strand breaks and if the DNA damage is not repaired properly, chromosome translocations and aneuploidy would occur [4]. The findings suggest, but do not prove, that, upon exposure to benzene, embryonic hematopoietic stem cells may not be able to effectively activate the defense system to defend against the toxicity of benzene metabolites in comparison with adult bone marrow hematopoietic stem cells and, therefore, are more likely to have gene-duplicating mutations and chromosome-specific translocations giving rise to leukemic clones. Further studies are needed to determine the role of Cyp4F18 and DNA-PKcs in these hematopoietic stem cells response to HQ toxicity as well as to further elucidate mechanisms responsible for the differential toxic responses to HQ in the two HSC populations.

We demonstrated that the benzene metabolite hydroquinone causes toxicity in two kinds of hematopoietic stem cells in mice. We compared the toxic responses to HQ between YS-HSC and BM-HSC from the aspects of cell proliferation, differentiation and apoptosis. Quantification of mRNA levels of Cyp4f18 and DNA-PKcs by quantitative real-time-PCR revealed the differential expressions of the two benzene-induced genes in YS-HSC and BM-HSC in response to hydroquinone. Our studies on the benzene metabolite-induced toxicity in the embryonic hematopoietic stem cells may be useful in understanding the pathogenesis of childhood leukemia. Our research provides a new experimental system for the assessment of the hematopoietic toxicity or embryotoxicity of environmental and therapeutic agents that potentially cause hematotoxicity or leukemia in children.
